# Remote liver ischemic preconditioning protects against renal ischemia/reperfusion injury via phosphorylation of extracellular signal-regulated kinases 1 and 2 in mice

**DOI:** 10.1371/journal.pone.0308977

**Published:** 2024-08-19

**Authors:** Qifeng Wang, Junshen Xiao, Shichao Wei, Xi Yang, Jiaxue Li, Yunxia Zuo, Zhaoyang Hu

**Affiliations:** 1 Department of Anesthesiology, West China Hospital, Sichuan University, Chengdu, Sichuan, China; 2 Laboratory of Anesthesia and Critical Care Medicine, National-Local Joint Engineering Research Centre of Translational Medicine of Anesthesiology, West China Hospital, Sichuan University, Chengdu, Sichuan, China; Gifu University School of Medicine Graduate School of Medicine: Gifu Daigaku Igakubu Daigakuin Igakukei Kenkyuka, JAPAN

## Abstract

Perioperative acute kidney injury (AKI), which is mainly mediated by renal ischemia‒reperfusion (I/R) injury, is commonly observed in clinical practice. However, effective measures for preventing and treating this perioperative complication are still lacking in the clinic. Thus, we designed this study to examine whether remote liver ischemic preconditioning (RLIPC) has a protective effect on damage caused by renal I/R injury. In a rodent model, 30 mice were divided into five groups to assess the effects of RLIPC and ERK1/2 inhibition on AKI. The groups included the sham-operated (sham), kidney ischemia and reperfusion (CON), remote liver ischemic preconditioning (RLIPC), CON with the ERK1/2 inhibitor U0126 (CON+U0126), and RLIPC with U0126 (RLIPC+U0126). RLIPC consisted of 4 liver ischemia cycles before renal ischemia. Renal function and injury were assessed through biochemical assays, histology, cell apoptosis and protein phosphorylation analysis. RLIPC significantly mitigated renal dysfunction, tissue damage, inflammation, and apoptosis caused by I/R, which was associated with ERK1/2 phosphorylation. Furthermore, ERK1/2 inhibition with U0126 negated the protective effects of RLIPC and exacerbated renal injury. To summarize, we demonstrated that RLIPC has a strong renoprotective effect on kidneys post I/R injury and that this effect may be mediated by phosphorylation of ERK1/2.

## Introduction

Perioperative acute kidney injury (AKI) is commonly observed in clinical practice. However, effective measures for preventing and treating this essential complication are still lacking [1]. Patients with sepsis and AKI may have an associated mortality rate of 70% [2]. In addition, AKI may reduce the function of other organ systems, which further leads to multiorgan failure, sepsis or death [3]. Therefore, there is a strong need for additional investigations to provide a thorough understanding of this pathology and efficient ways to assist patient recovery.

Renal ischemia/reperfusion (I/R) injury, one of the main pathological mechanisms of AKI, has been widely investigated as a potential target for treating AKI [4]. Studies have shown that various measures can be taken to alleviate renal I/R injury, including the inhibition of Brd4 with JQ1 [5], carbon monoxide [6] and miR-20a-5p [7]. Remote ischemic preconditioning (RIPC), such as remote liver ischemic preconditioning (RLIPC), has attracted the attention of many researchers because of its simplicity and lack of side effects [8]. Our previous studies also demonstrated its protective effect on sudden cardiac death [9] and myocardial I/R injury in streptozotocin-induced diabetic rats [10]. Most importantly, a recent clinical study comparing RIPC versus local ischemic preconditioning of the liver also demonstrated the positive effect of this treatment [11]. However, whether this technique has a protective effect on renal injury caused by I/R has not been determined. Moreover, the mechanism behind this technique is worth exploring.

In this study, we performed an animal study based on a previously reported rodent model to investigate the renoprotective role of RLIPC and its underlying mechanism. Our research provides a new idea for the treatment of patients with this pathological change in clinical practice, and also provides a reference for the development of related targeted drugs.

## Materials and methods

### Ethical approval

Our research adhered to the principles outlined in the "Guide for the Care and Use of Laboratory Animals" (Eighth edition, 2011). The study protocols received approval from the Institutional Animal Care and Use Committee of Sichuan University, Chengdu, Sichuan, China (Approval No: 20211396A).

### Animals

C57BL/6 mice, obtained from Chengdu Dashuo Experimental Animal Co., Ltd. (Chengdu, China), with a body weight ranging from 20 to 30 g and an average age of 10 months, were utilized in this investigation. All mice were provided ad libitum access to water and food. The experiments were conducted on mice of both sexes. The mice were housed in a specific-pathogen-free animal facility with a circadian rhythm of 12 hours light and 12 hours darkness.

### Study design

**Fig 1:**
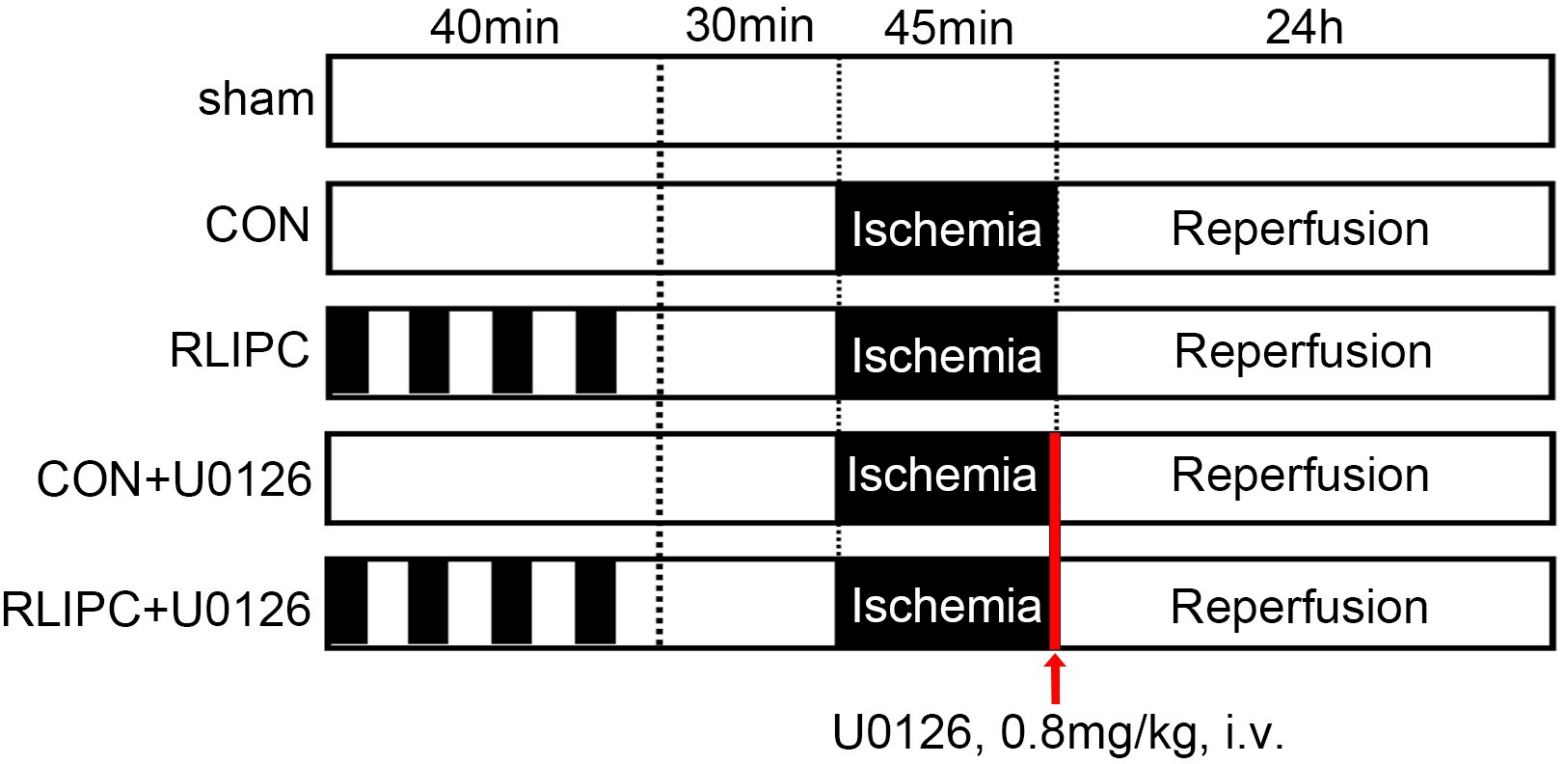
Animal experiment protocols. All groups (except for the sham-operated mice) underwent renal ischemia injury induced by bilateral clamping of the renal pedicles for 45 minutes. After renal ischemia, the animals were allowed to recover from anesthesia and were allowed 24 hours of reperfusion. RLIPC treatment comprising of four cycles of 5 min of liver ischemia and 5 min of reperfusion stimuli was conducted 30 min before renal ischemia in RLIPC and RLIPC+U0126 groups. The ERK1/2 inhibitor U0126 was intravenously administered to the mice after the removal of clamps in CON+U0126 and RLIPC+U0126 groups, while 0.9% NaCl in other groups.

Animals were randomly assigned to 5 treatment groups, as shown in Fig 1: (1) The sham-operated group (sham, n = 6) received only surgical laparotomy procedures; (2) the control group (CON, n = 6) received I/R treatment; (3) the remote liver ischemic preconditioning group (RLIPC, n = 6) received both I/R and RLIPC treatment; (4) the control + inhibitor group (CON+U0126, n = 6) received U0126 injection based on protocols in CON group; and (5) the RLIPC + inhibitor group (RLIPC+U0126, n = 6) received U0126 injection based on protocols in RLIPC group. The kidneys of the sham-operated animals underwent identical surgical laparotomy procedures except for occlusion of the renal pedicles. Kidneys in the other four groups were subjected to 45 min of ischemia and 24 hours of reperfusion. U0126, a specific ERK1/2 inhibitor (0.8 mg/kg; MedChemExpress, Monmouth Junction, NJ, USA), was given to the mice intravenously in the CON+U0126 and RLIPC+U0126 groups from the femoral vein soon after removal of clamps, while physiological saline (0.9% NaCl) solution was given the same way in other groups as negative control. No statistical method was applied to calculate the sample size. Instead, it was determined mainly based on results from identical experimental models in the literature and from measurements.

### RLIPC

To implement RLIPC treatment, mice were anesthetized and received similar surgical procedures as mice in CON or CON+U0126 group. Seventy minutes before renal ischemia, hepatic pedicle of mice in RLIPC and RLIPC+U0126 groups were ligated for 5 min, followed by 5 min of reperfusion as a cycle. The cycle was repeated for four times to facilitate the protective effect. Mice were left with a 30 min rest period before renal ischemia to stabilize circulation.

### Mouse model of renal I/R injury

To explore the protective effects of RLIPC on renal I/R injury, we established a mouse model based on our prior expertise [10, 12]. In summary, a model of renal I/R injury was induced by bilaterally clamping the renal pedicles for 45 minutes using microaneurysm clamps (Dieffenbach Bulldog Clamps, Harvard Apparatus Ltd.) under general anesthesia with phenobarbital sodium (50 mg/kg, i.p.) under aseptic conditions. Blood flow normalization in the kidneys was assessed at the onset of reperfusion through the observed color change, indicating the return to a red hue. Before surgery started, the mice were ensured to be adequately anesthetized by testing the pedal withdrawal reflex and lack of eye blink reflex. Analgesia was administered subcutaneously with nalbuphine (2 mg/kg). The temperature of the mice was carefully maintained at 37°C throughout the experiment using a heating pad. Following recovery from anesthesia, the animals were returned to their previous environment and allowed 24 hours of reperfusion.

### Sample collection

For sample collection, animals were intraperitoneally administered pentobarbital sodium (200mg/kg). Following euthanasia, plasma samples were obtained via heart puncture using heparin-containing tubes and subsequently centrifuged (1000 g, 10 min, 4°C) for subsequent biochemical analyses. The two kidneys of each mouse were either snap-frozen in liquid nitrogen and stored at -80°C for protein quantification or fixed with 4% paraformaldehyde overnight after the kidney weight (KW) was measured. Tibial length (TL) was measured to normalize KW. To mitigate selection bias and potential confounding factors, samples from each animal in the designated groups were collected and analyzed under uniform conditions.

### Renal function assessment

To assess renal function of the animals in each group, we quantified the plasma concentrations of creatinine (CREA) and urea (UREA) using a Mindray BS-120 Chemistry Analyzer (Mindray Medical, Shenzhen, China).

### Morphological analysis

To evaluate morphological changes after renal I/R injury and investigate the protective effects of RLIPC on kidney injury, hematoxylin& eosin (HE) staining and alcian blue-periodic acid-Schiff (PAS) staining (Solarbio, Beijing, China) were conducted following methodologies established in previous reports with slight modifications [13]. Subsequently, the sections were scrutinized, and 10 randomly chosen fields were imaged for each section using an inverted microscope (CAST system, Olympus A/S, Ballerup, Denmark). Two pathologists independently scored each image or field in a double-blinded fashion, focusing on parameters such as glomerular necrosis, intraluminal casts, and tubular necrosis. The detailed scoring methods were as follows [12, 14]:

**Table:** 

Score	Definition
0	no injury
1	injured area less than 25%
2	injured area between 25% and 50%
3	injured area between 50% and 75%
4	injured area more than 75%

### Apoptosis evaluation

Apoptotic cells in renal tissues were evaluated through the terminal deoxynucleotidyl transferase (TdT) dUTP nick-end labeling (TUNEL) assay. An In Situ Cell Death Detection Kit (DeadEnd™ Fluorometric TUNEL system, Promega Corporation, Madison, WI, USA) was utilized following the manufacturer’s instructions. The average percentage of TUNEL-positive cells relative to the total nuclei population was determined by counting 10 randomly selected fields under a Nikon fluorescence microscope (Eclipse Ni-E, Nikon, Tokyo, Japan).

### Western blot analysis

Kidney tissues were rinsed with ddH_2_O and homogenized in ice-cold RIPA buffer comprising 50 mM Tris–HCl (pH 7.4), 150 mM NaCl, 1% NP-40, 1 mM EDTA, 0.25% sodium deoxycholate, a phosphatase inhibitor cocktail, and a protease inhibitor cocktail (Sigma Chemical Co., St. Louis, MO, USA). The tissues were mechanically ground using a manual grinder on ice (Fisher Scientific, Hampton, NH, USA), followed by centrifugation at 10,000 × g for 10 minutes at 4°C. The protein concentration was determined using the BCA method (Pierce, Rockford, IL, USA). Subsequently, the proteins were separated via 12% Tris-glycine SDS‒PAGE and transferred to a nitrocellulose blotting membrane (Pall Corporation, Pensacola, FL, USA). The membranes were then blocked with a solution of 5% nonfat milk dissolved in PBST (PBS containing 0.1% Tween 20). The primary antibodies utilized in this study included antibodies against phosphorylated ERK1/2 (Thr202/Tyr204) (p-ERK1/2), total ERK1/2 (ERK1/2), and phosphorylated STAT-3 (Tyr705) (p-STAT-3); total STAT-3 (STAT-3); phosphorylated AKT (Ser473) (p-AKT); and total AKT (AKT) (all 1:1000; sourced from Cell Signaling Technology, Danvers, MA, USA). A peroxidase-conjugated goat anti-rabbit secondary antibody (1:5000; Bio-Rad, Hercules, CA, USA) and super ECL detection reagent (YEASEN Bio. Inc., Shanghai, China) were utilized. ImageJ (National Institutes of Health, Bethesda, MD, USA) was used for quantitative analysis.

### Immunohistochemical analysis

Immunohistochemical analysis was used to evaluate protein expression according to our protocol. Briefly, kidney tissue was immediately fixed in 4% paraformaldehyde. After embedding, the kidneys were sliced into 4 μm thick sections. Dewaxing and rehydration of sections, antigen retrieval, and antigen blocking with peroxidase were performed in sequence. Next, primary antibodies against phosphorylated ERK1/2 (p-ERK1/2, 1:200; Cell Signaling Technology, Danvers, MA, USA), IL-6, TNF-α, NGAL, Bcl-2, and Bax (all 1:200; Affinity Biosciences, Cincinnati, OH, USA) were incubated with the endogenous proteins at 4°C overnight. The secondary antibody used was a peroxidase-conjugated goat anti-rabbit antibody (Santa Cruz Biotechnology, Santa Cruz, CA) diluted in phosphate-buffered saline (1:250). The renal slices were then incubated with this antibody for 30 minutes at 37°C and subsequently treated with 3,3’-diaminobenzidine (DAB; Beijing Zhongshan Golden Bridge Biotechnology, Beijing, China). Images were captured using an inverted microscope (CAST system, Olympus A/S, Ballerup, Denmark). Ten random fields were selected from each slide. The mean intensity of the positively stained area was analyzed using Image-Pro Plus software (Media Cybernetics, Inc., Carlsbad, CA, USA).

### Statistical analysis

The data are expressed as mean ± standard deviation (SD). Statistical analysis was performed using GraphPad Prism 8 (GraphPad Software, Inc., La Jolla, CA, USA) software. One-way analysis of variance (ANOVA) was used to analyze the data. The equality of variances was examined using Levene’s test. Between-group comparisons were conducted using the Student–Newman–Keuls test if the variances were equal. Otherwise, Dunnett’s T3 test was applied. A *P* value of less than 0.05 was considered to indicate statistical significance. No intentional exclusion of samples or animals was made in the analyses. Although our experiments were not completely blinded, we followed standard laboratory procedures of randomization and treated the data in a blinded manner.

## Results

### RLIPC alleviates renal injury after I/R

**Fig 2:**
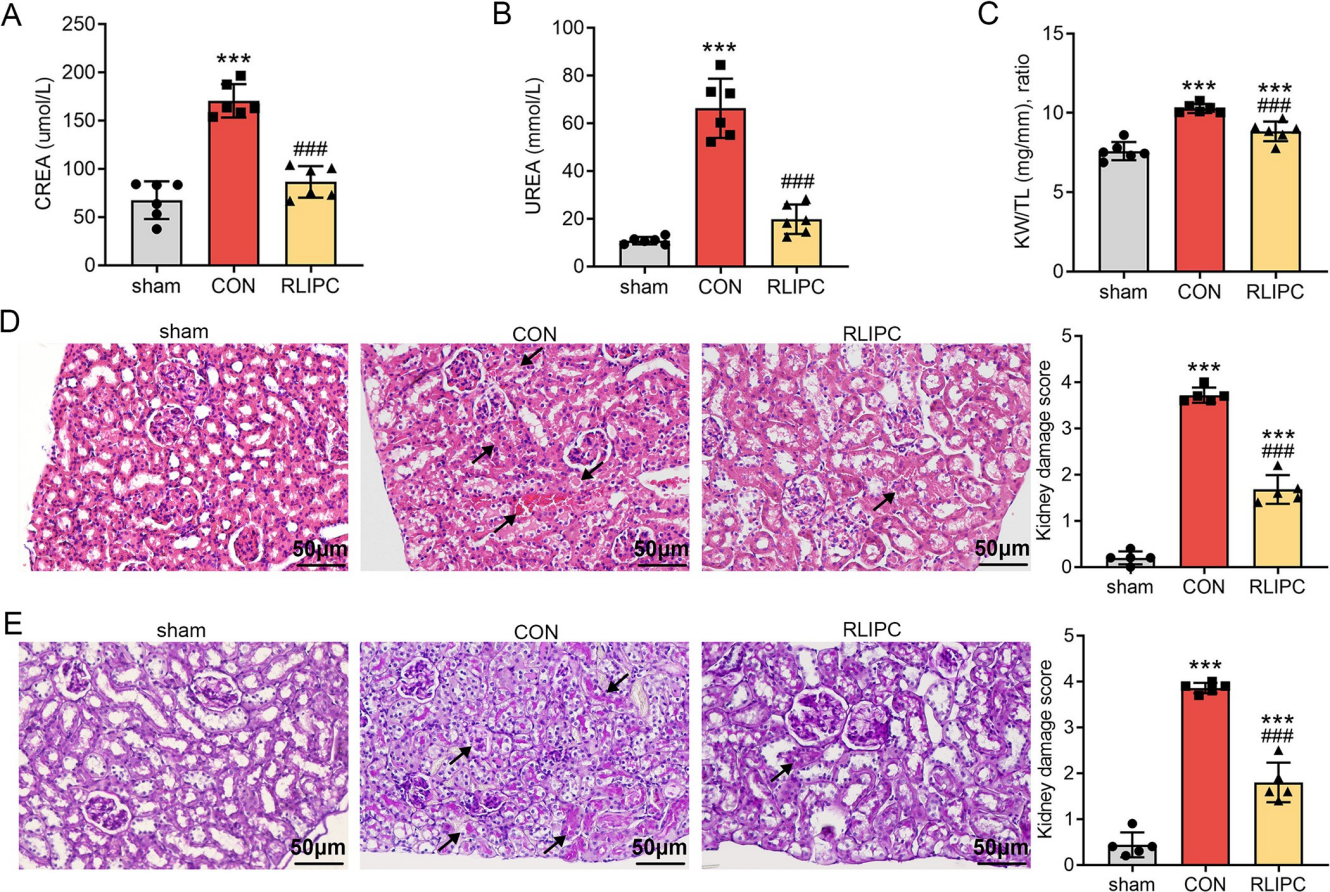
RLIPC alleviates renal injury after I/R. A-B. Plasma levels of creatinine (CREA) (A) and urea (UREA) (B) in mice subjected to 45 min of renal ischemia followed by 24 h of reperfusion. ***P<0.001, compared with sham mice; ###P<0.001, compared with CON mice. n = 6 per group. sham: sham-operated, CON: control, RLIPC: remote liver ischemic preconditioning. C. Kidney weight-to-tibia length (KW/TL) ratios of the mice. n = 6 per group. ***P<0.001, compared with sham mice; ###P<0.001, compared with CON mice. sham: sham-operated, CON: control, RLIPC: remote liver ischemic preconditioning. D. Left, Representative images of kidney sections stained with HE after renal I/R injury. Scale bars, 50 μm. Black arrows indicate injuries. Right, Differences between the kidney damage scores of the groups. n = 5 per group. ***P<0.001, versus sham mice; ###P<0.001, versus CON mice. sham: sham-operated, CON: control, RLIPC: remote liver ischemic preconditioning. E. Left, Representative kidney sections stained with PAS after renal I/R injury. Scale bars, 50 μm. Black arrows indicate injuries. Right, Kidney damage scores in each group. n = 5 per group. ***P<0.001, versus sham mice; ###P<0.001, versus CON mice. sham: sham-operated, CON: control, RLIPC: remote liver ischemic preconditioning.

Whether RLIPC, a widely examined protective strategy in I/R injury [15–17], can protect kidneys from I/R injury is still unknown. Here, we first evaluated the levels of creatinine (CREA) and urea (UREA) in mouse plasma. Biochemical analysis revealed that both CREA (170.5±17.3μmol/L, *P<0*.*001* vs. sham) and UREA (66.3±12.4 mmol/L, *P<0*.*001* vs. sham) increased significantly in the CON group, which suggested that renal I/R treatment leads to reduced renal function. However, RLIPC dramatically reversed both CREA (86.5±16.3μmol/L, *P<0*.*001* vs. CON) and UREA (19.8±6.2 mmol/L, *P<0*.*001* vs. CON) levels, indicating the renoprotective effects of this strategy (Fig 2A and 2B). Kidney swelling was quantified by the average kidney weight-to-tibia length (KW/TL) ratio, which indicates evidence of significant tubular injury. Our results showed that mice with kidney I/R damage had a greater KW/TL ratio (10.3±0.3, *P<0*.*001* vs. sham), while mice treated with RLIPC (8.8±0.6, *P<0*.*001* vs. CON) had a lower KW/TL ratio than did the CON group (Fig 2C). To assess the effects of RLIPC on renal morphology, we performed HE staining and found that kidneys in the CON group were significantly damaged, and this change was accompanied by glomerular cell fragmentation, destruction of renal tubule structure, protein cast formation and swelling, whereas kidneys in the RLIPC group exhibited fewer damage morphologies. Quantitative analysis of kidney damage revealed a score of 3.7±0.2 (*P<0*.*001* vs. sham) in the CON group and 1.7±0.3 (*P<0*.*001* vs. CON) in the RLIPC group (Fig 2D). The PAS staining results were also in line with these observations (*P<0*.*001* vs. CON; Fig 2E). Taken together, these findings demonstrated the renoprotective effects of RLIPC on renal I/R-induced injury.

### RLIPC alleviates the I/R-induced renal inflammatory response

**Fig 3:**
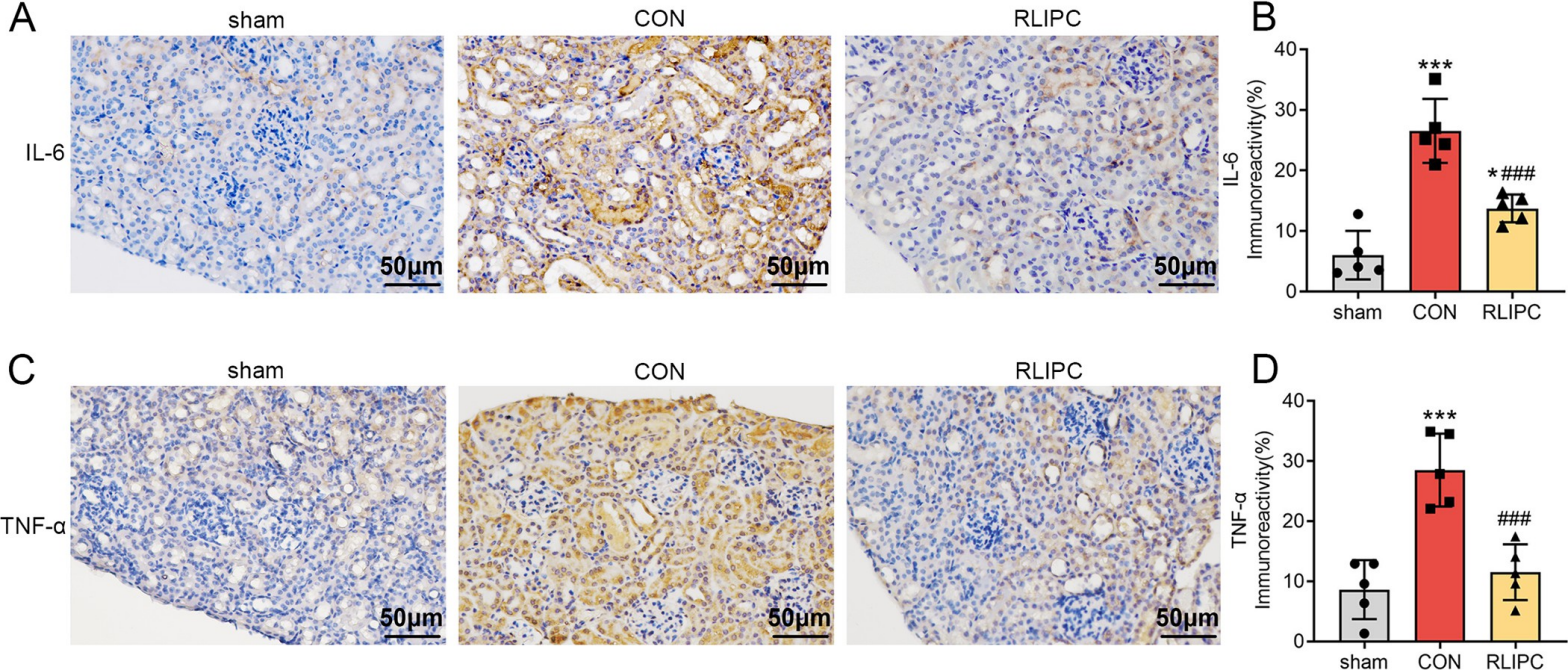
RLIPC alleviates the I/R-induced renal inflammatory response. A. Representative images of kidney sections immunostained for interleukin-6 (IL-6) after renal I/R injury. Scale bars, 50 μm. sham: sham-operated, CON: control, RLIPC: remote liver ischemic preconditioning. B. Levels of IL-6 immunoreactivity in mouse kidneys post I/R. n = 5 per group. *P<0.05, ***P<0.001, versus sham mice; ###P<0.001, versus CON mice. sham: sham-operated, CON: control, RLIPC: remote liver ischemic preconditioning. C. Representative images of kidney sections immunostained for tumor necrosis factor-α (TNF-α) after renal I/R injury. Scale bars, 50 μm. sham: sham-operated, CON: control, RLIPC: remote liver ischemic preconditioning. D. TNF-α immunoreactivity levels in mouse kidneys post I/R. n = 5 per group. ***P<0.001, versus sham mice; ###P<0.001, versus CON mice. sham: sham-operated, CON: control, RLIPC: remote liver ischemic preconditioning.

Interleukin-6 (IL-6) and tumor necrosis factor-α (TNF-α) are widely used inflammatory biomarkers in medical research. Herein, we performed immunohistochemical staining to investigate the inflammatory response post I/R and the effects of RLIPC on this response using these two markers. As shown in Fig 3A and 3B, I/R injury significantly promoted the expression of IL-6 within renal tissues (26.5±5.3%, *P<0*.*001* vs. sham), indicating a stronger inflammatory response. In contrast, RLIPC treatment significantly downregulated the expression level of IL-6 (13.7±2.3%, *P<0*.*001* vs. CON). In accordance with these findings, the expression of TNF-α was also upregulated in the CON group (28.5±6.0%, *P<0*.*001* vs. sham), and the RLIPC strategy significantly decreased the TNF-α protein level (11.5±4.6%, *P<0*.*001* vs. CON; Fig 3C and 3D). These results suggest that RLIPC may alleviate renal I/R injury by inhibiting the inflammatory response.

### RLIPC downregulates NGAL expression in kidneys with I/R injury

**Fig 4:**
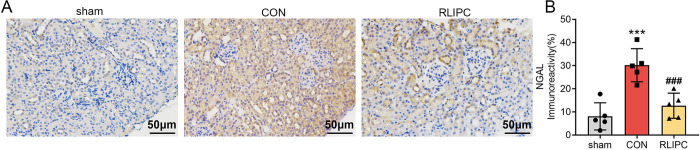
Effect of RLIPC on renal NGAL expression levels after renal I/R injury. A. Representative images of kidney sections immunostained for neutrophil gelatinase-associated lipocalin (NGAL) after renal I/R injury. Scale bars, 50 μm. sham: sham-operated, CON: control, RLIPC: remote liver ischemic preconditioning. B. NGAL immunoreactivity levels in mouse kidneys post I/R. n = 5 per group. ***P<0.001, versus sham mice; ###P<0.001, versus CON mice. sham: sham-operated, CON: control, RLIPC: remote liver ischemic preconditioning.

Neutrophil gelatinase-associated lipocalin (NGAL), a new tubule injury marker in AKI [18], was evaluated in our study to further determine the renoprotective role of RLIPC after renal I/R attack. Immunohistochemical staining revealed that NGAL expression was significantly greater in CON-treated mice than in sham-treated mice (30.2±7.2%, *P<0*.*001*). As expected, RLIPC treatment drastically decreased the expression of this protein compared to that in the untreated CON group (12.7±5.4%, *P<0*.*001* vs. CON; Fig 4A and 4B). These findings provide additional evidence of the renoprotective effects of RLIPC in an animal model.

### RLIPC inhibits renal apoptosis induced by I/R injury

**Fig 5:**
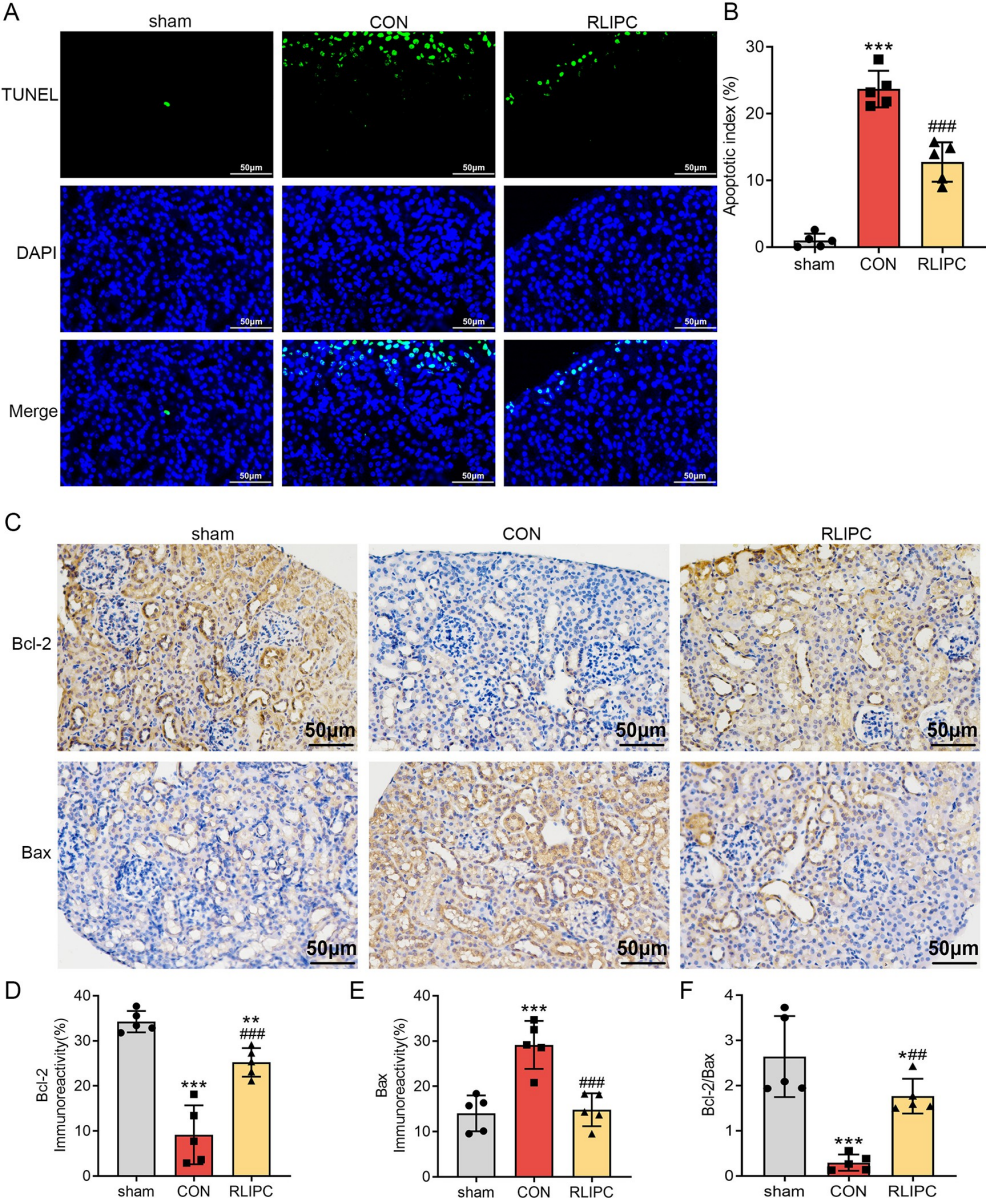
RLIPC inhibits renal apoptosis post-I/R. A. Representative images of TUNEL staining of mouse kidney sections. Scale bars, 50 μm. TUNEL-positive cells were stained green, and nuclei were stained with DAPI (blue). sham: sham-operated, CON: control, RLIPC: remote liver ischemic preconditioning. B. The ratio of TUNEL-positive cells. n = 5 per group. ***P<0.001, versus sham mice; ###P<0.001, versus CON mice. sham: sham-operated, CON: control, RLIPC: remote liver ischemic preconditioning. C. Representative images of immunostaining for Bcl-2 and Bax in kidney sections after renal I/R injury. sham: sham-operated, CON: control, RLIPC: remote liver ischemic preconditioning. D-F. The analysis of Bcl-2 (D), Bax (E) and Bcl-2/Bax (F) protein expression in mouse kidneys post-I/R. n = 5 per group. *P<0.05, **P<0.01, ***P<0.001, versus sham mice; ##P<0.01, ###P<0.001, versus CON mice. sham: sham-operated, CON: control, RLIPC: remote liver ischemic preconditioning.

Next, we performed a TUNEL assay to examine whether RLIPC can inhibit renal apoptosis caused by I/R injury. Apoptotic cells were stained green as positive, and the nuclei were stained blue. The ratio of green-stained to blue-stained cells was calculated in this study to assess renal apoptosis. We showed that I/R can cause severe renal apoptosis (23.7±2.7%, *P<0*.*001* vs. sham). Notably, RLIPC strongly inhibited renal apoptosis caused by I/R (12.7±3.0%, *P<0*.*001* vs. CON; Fig 5A and 5B). In line with these observations, immunohistochemical staining of apoptotic-related proteins including Bcl-2 (anti-apoptotic protein) and Bax (pro-apoptotic protein) both provided supporting evidence (Fig 5C), with Bcl-2 downregulated in CON mice (9.2±6.5%, *P<0*.*001* vs. sham) and increased to higher level in RLIPC group mice (25.2±3.2%, *P<0*.*001* vs. CON, Fig 5D), Bax upregulated in CON mice (29.2±5.3%, *P<0*.*001* vs. sham) and becoming lower in RLIPC-treated mice (14.8±3.6%, *P<0*.*001* vs. CON, Fig 5E). In addition, a greater ratio of Bcl-2 to Bax was detected in RLIPC-operated mice (1.8±0.4, *P<0*.*01* vs. CON; Fig 5F). Taken together, these findings indicated that RLIPC may also protect against renal I/R injury by inhibiting apoptosis.

### RLIPC promotes ERK1/2 phosphorylation following renal I/R

**Fig 6:**
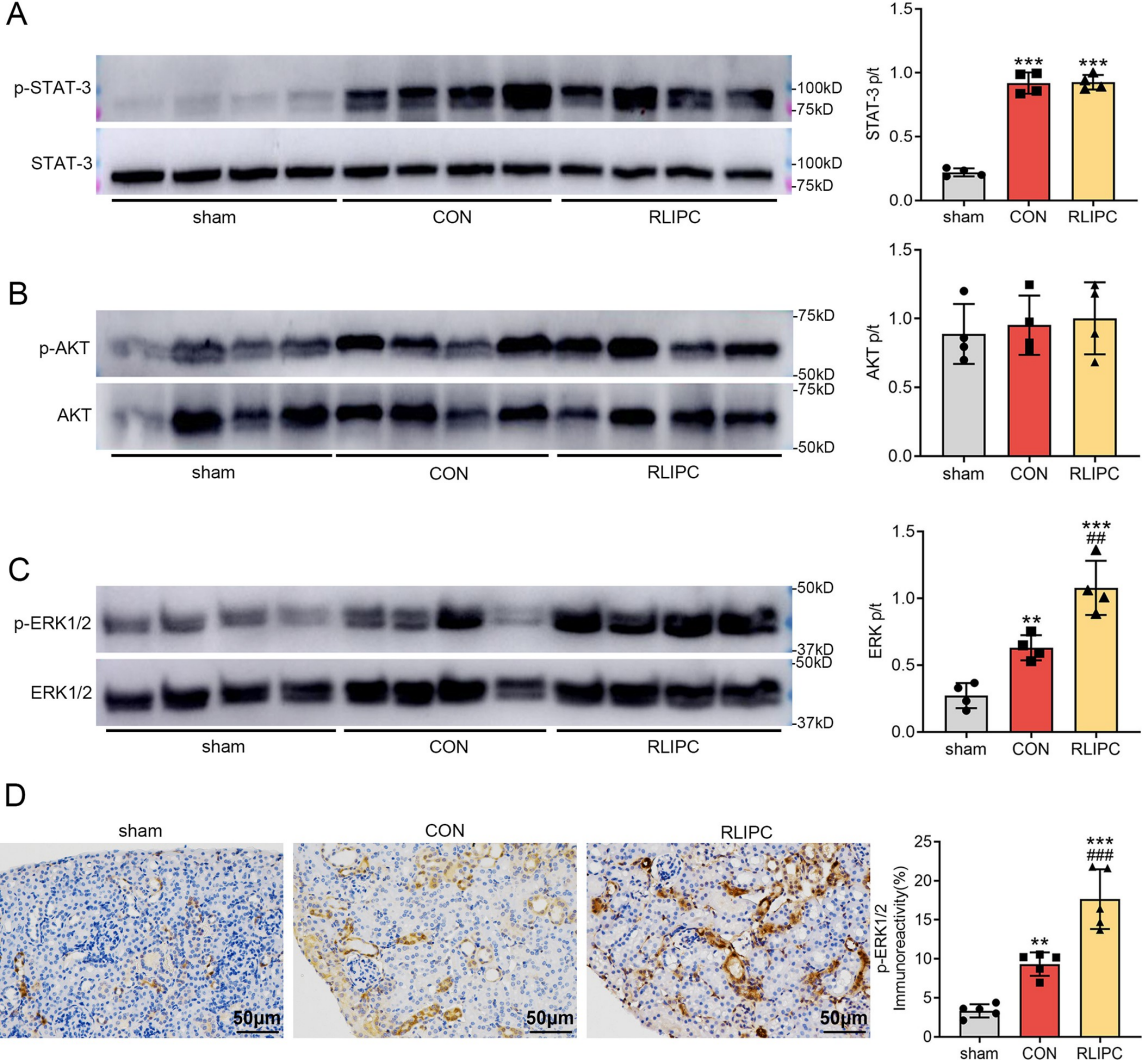
Effect of RLIPC on renal ERK1/2 phosphorylation post-I/R. A-C. Left, Representative western blots of renal p-STAT-3 and STAT-3 (A), p-AKT and AKT (B), and p-ERK1/2 and ERK1/2 (C) after I/R injury. Right, the relative abundance of phosphorylated proteins was normalized to the total protein concentration. n = 4 per group. **P<0.01, ***P<0.001, versus sham mice; ##P<0.01, versus CON mice. sham: sham-operated, CON: control, RLIPC: remote liver ischemic preconditioning. D. Left, Representative images of kidney sections immunostained for p-ERK1/2 after renal I/R injury. Scale bars, 50 μm. Right, p-ERK1/2 immunoreactivity levels. n = 5 per group. **P<0.01, ***P<0.001, versus sham mice; ###P<0.001, versus CON mice. sham: sham-operated, CON: control, RLIPC: remote liver ischemic preconditioning.

We and others have shown that the reperfusion injury salvage kinase (RISK) pathway and the survivor activating factor enhancement (SAFE) pathway both play key roles in I/R injury [12, 19–21]. However, whether these two pathways mediate the renoprotective effect of RLIPC on kidneys post I/R has not been determined. Here, we conducted western blot experiments on several proteins in these signaling pathways. We found that I/R promoted the phosphorylation of STAT-3 (0.9±0.1, *P<0*.*001* vs. sham) and ERK1/2 (0.6±0.1, *P<0*.*01* vs. sham) in the CON group, whereas the phosphorylation of AKT remained constant between the sham and CON groups (*P>0*.*05* vs. sham). Most importantly, compared with CON mouse kidneys, RLIPC further phosphorylated ERK1/2 (1.0±0.1, *P<0*.*01* vs. CON), but had no effect on the other proteins (*P>0*.*05* vs. CON; Fig 6A to 6C). To further confirm these findings, we performed an immunohistochemistry staining and found that the expression of phosphorylated ERK1/2 increased significantly in the kidneys of the CON mice (9.3±1.5%, *P<0*.*01* vs. sham). Furthermore, compared with I/R-operated kidneys, RLIPC therapy dramatically increased the expression of phosphorylated ERK1/2 (17.7±3.8%, *P<0*.*001*; Fig 6D). Overall, we found that RLIPC may protect kidneys against I/R injury via phosphorylation or activation of ERK1/2.

### U0126 eliminates the protective effects of RLIPC on I/R-injured mouse kidneys

**Fig 7:**
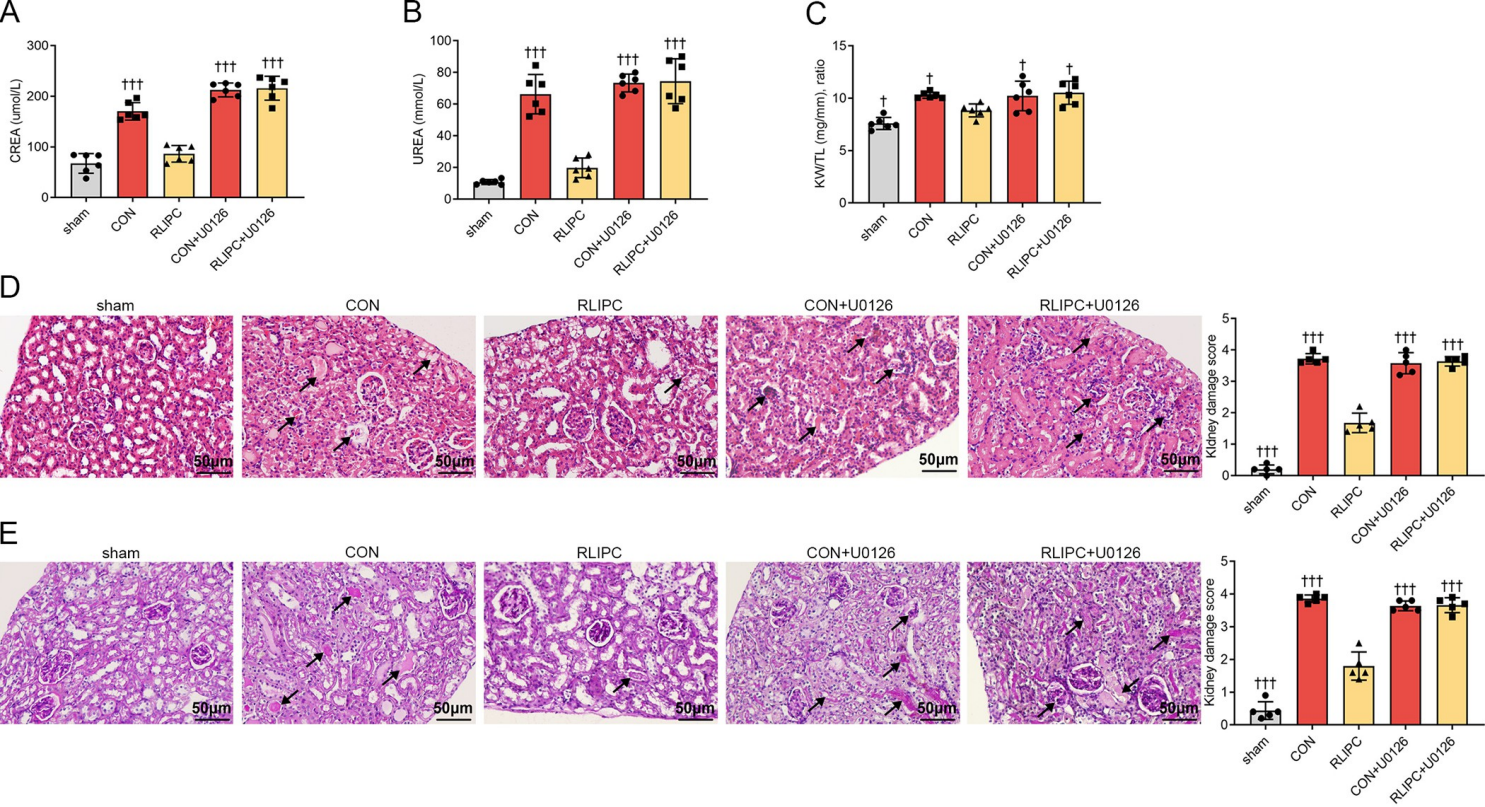
Pharmacological inhibition of ERK1/2 reversed the renal protection induced by RLIPC. A-B. Plasma levels of creatinine (CREA, A) and urea (UREA, B) in mice treated with or without U0126. †††P<0.001, compared with RLIPC mice. n = 6 per group. sham: sham-operated, CON: control, RLIPC: remote liver ischemic preconditioning, CON+U0126: control+U0126, RLIPC+U0126: remote liver ischemic preconditioning+U0126. The values for the sham, CON and RLIPC mice are repeated from Fig 2A and 2B for comparison. C. Kidney weight-to-tibia length (KW/TL) ratios of the mice treated with or without U0126. n = 6 per group. †P<0.05, compared with RLIPC mice. The values for the sham, CON and RLIPC mice are repeated from Fig 1E for comparison. sham: sham-operated, CON: control, RLIPC: remote liver ischemic preconditioning, CON+U0126: control+U0126, RLIPC+U0126: remote liver ischemic preconditioning+U0126. The values for the sham, CON and RLIPC mice are repeated from Fig 2C for comparison. D-E. Left, Representative images of kidney sections stained with HE (D) or PAS (E) in the presence or absence of U0126 after renal I/R. sham: sham-operated, CON: control, RLIPC: remote liver ischemic preconditioning, CON+U0126: control+U0126, RLIPC+U0126: remote liver ischemic preconditioning+U0126. Scale bars, 50 μm. Black arrows indicate injuries. Right, Kidney damage scores in each group. n = 5 per group. †††P<0.001, compared with RLIPC mice. The values for the sham, CON and RLIPC mice are repeated from Fig 2D and 2E for comparison.

To further test the role of ERK1/2 phosphorylation and activation in RLIPC-mediated renal protection, we used U0126, a specific antagonist of ERK1/2 activation. We administered U0126 through femoral vein injection at a dosage of 0.8 mg/kg at the onset of renal reperfusion. As shown in Fig 7A, the administration of U0126 significantly increased the CREA concentration in the CON+U0126 group (212.6±13.7μmol/L, *P<0*.*001* vs. RLIPC) and the RLIPC+U0126 group (215.9±23.6μmol/L, *P<0*.*001* vs. RLIPC) to a level post I/R. UREA also yielded similar results to those of the RLIPC group (all *P<0*.*001* vs. RLIPC; Fig 7B). In addition, the KW/TL ratios in the group treated with U0126 increased due to the inhibition of ERK1/2 phosphorylation (all *P<0*.*05* vs. RLIPC; Fig 7C). These results suggested that the protective effects of RLIPC on the kidney were eliminated by the inhibition of ERK1/2 phosphorylation, indicating the critical role of this protein regulation process. In accordance with the findings of the CREA and UREA results, the HE staining assays also showed that kidney tissue was more damaged in the CON+U0126 (3.58±0.33, *P<0*.*001*) and RLIPC+U0126 (3.64±0.15, *P<0*.*001*; Fig 7D) groups than in the RLIPC-treated group. Consistent with the HE staining assays results, PAS-stained sections also revealed more extensive damage in the kidneys of the CON+U0126 group (3.64±0.15, *P<0*.*001*) and RLIPC+U0126 group (3.66±0.23, *P<0*.*001*; Fig 7E).

**Fig 8:**
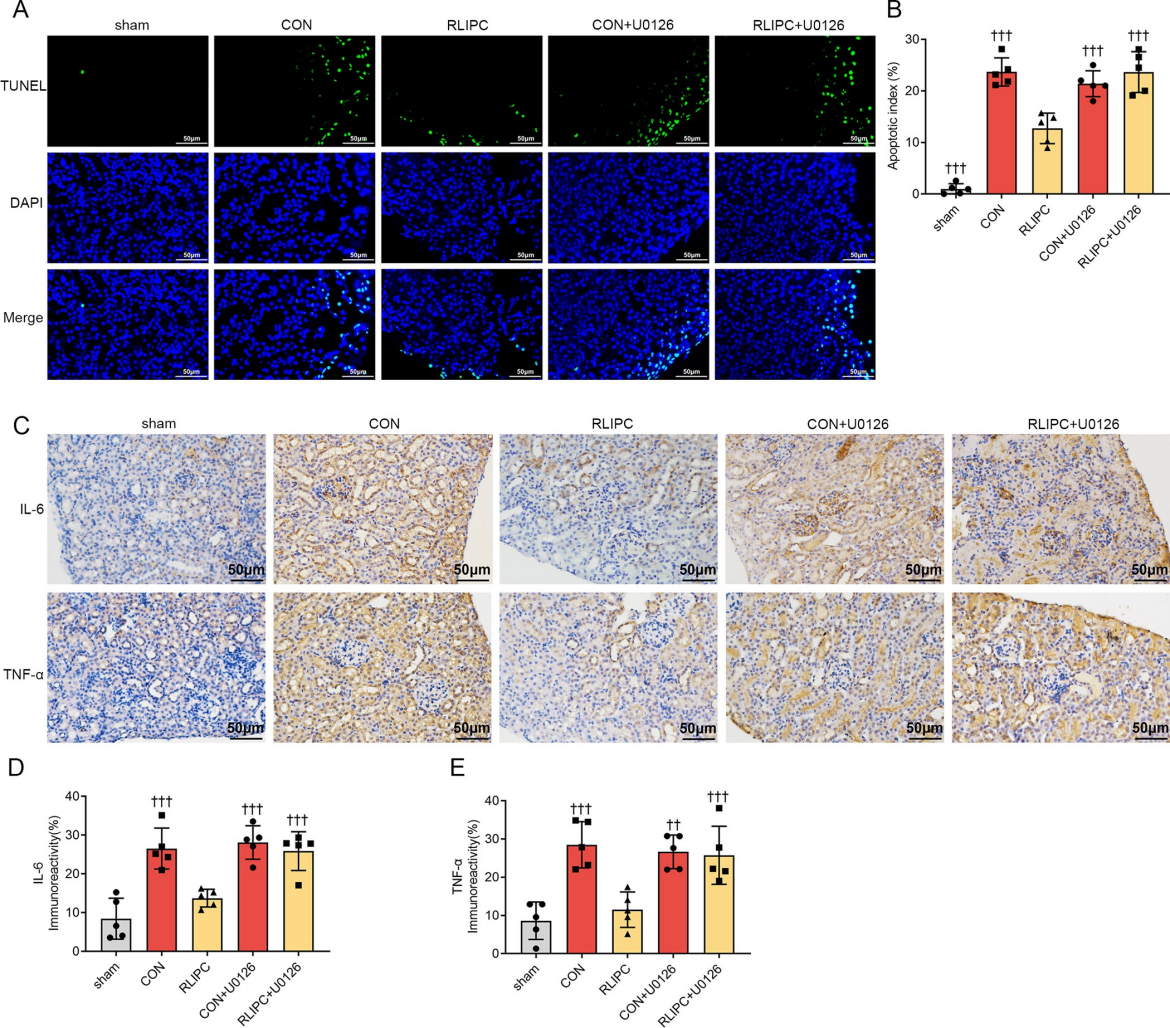
U0126 reversed RLIPC-induced renal apoptosis and inflammatory reduction after I/R injury. A. Representative images of renal apoptosis in the presence or absence of U0126 after renal I/R. sham: sham-operated, CON: control, RLIPC: remote liver ischemic preconditioning, CON+U0126: control+U0126, RLIPC+U0126: remote liver ischemic preconditioning+U0126. Scale bars, 50 μm. B. The ratio of TUNEL-positive cells. n = 5 per group. †††P<0.001, compared with RLIPC mice. sham: sham-operated, CON: control, RLIPC: remote liver ischemic preconditioning, CON+U0126: control+U0126, RLIPC+U0126: remote liver ischemic preconditioning+U0126. The values for the sham, CON and RLIPC mice are repeated from Fig 5B for comparison. C. Representative images of immunostaining for IL-6 and TNF-α in kidney sections in the presence or absence of U0126 after renal I/R injury. Scale bars, 50 μm. sham: sham-operated, CON: control, RLIPC: remote liver ischemic preconditioning, CON+U0126: control+U0126, RLIPC+U0126: remote liver ischemic preconditioning+U0126. D. Level of IL-6 immunoreactivity in mouse kidneys post I/R. n = 5 per group. †††P<0.001, compared with RLIPC mice. sham: sham-operated, CON: control, RLIPC: remote liver ischemic preconditioning, CON+U0126: control+U0126, RLIPC+U0126: remote liver ischemic preconditioning+U0126. The values for the sham, CON and RLIPC mice are repeated from Fig 3B for comparison. E. TNF-α immunoreactivity levels in mouse kidneys post I/R. n = 5 per group. ††P<0.01, †††P<0.001 versus RLIPC mice. sham: sham-operated, CON: control, RLIPC: remote liver ischemic preconditioning, CON+U0126: control+U0126, RLIPC+U0126: remote liver ischemic preconditioning+U0126. The values for the sham, CON and RLIPC mice are repeated from Fig 3D for comparison.

TUNEL assays also demonstrated that renal apoptosis was reversed in both the CON+U0126 (21.4±2.5%, *P<0*.*001* vs. RLIPC) and RLIPC+U0126 (23.7±4.0%, *P<0*.*001* vs. RLIPC) groups (Fig 8A and 8B), with more cells stained positive for apoptosis. Immunohistochemical analysis revealed that renal IL-6 was more highly secreted in both groups treated with U0126 (all *P<0*.*001* vs. RLIPC; Fig 8C and 8D). Moreover, immunostaining for TNF-α showed similar results (*P<0*.*01 or P<0*.*001* vs. RLIPC; Fig 8C and 8E).

**Fig 9:**
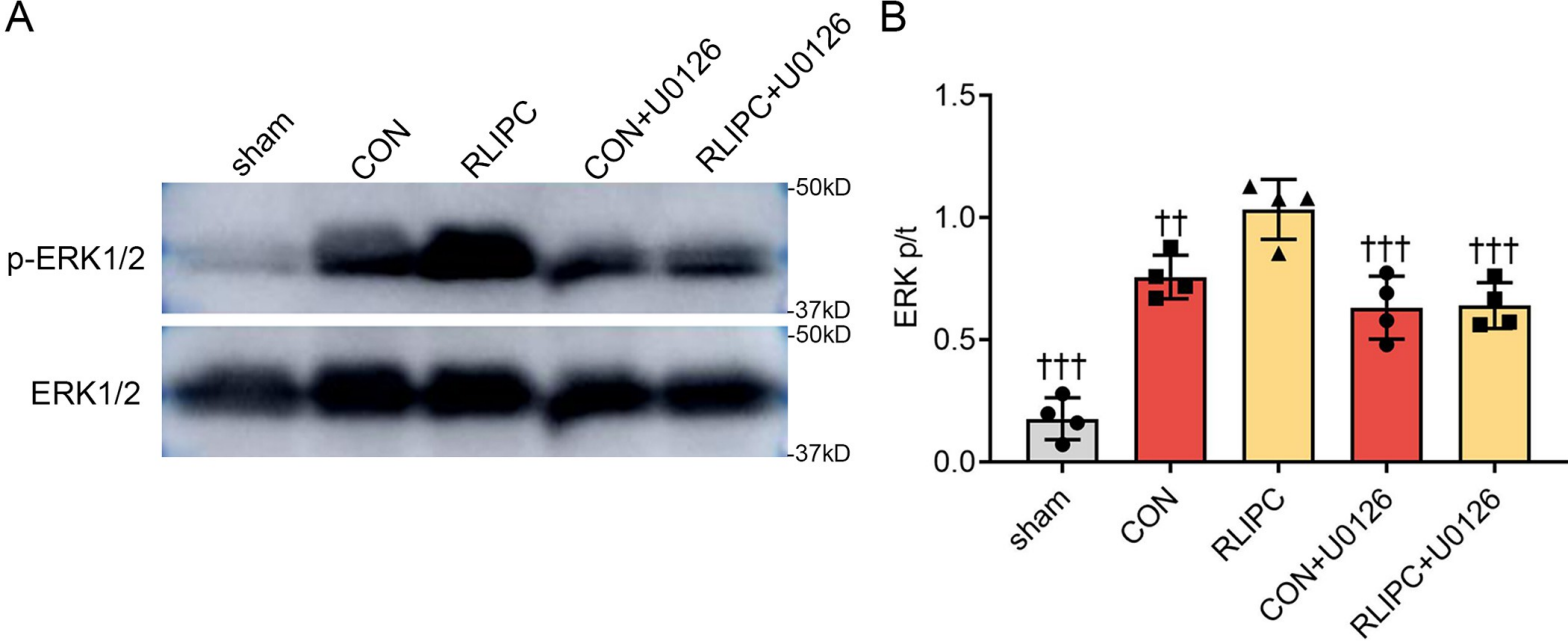
ERK1/2 phosphorylation after U0126 treatment. A. Representative western blot of renal p-ERK1/2 and ERK1/2 in the presence or absence of U0126 after I/R injury. sham: sham-operated, CON: control, RLIPC: remote liver ischemic preconditioning, CON+U0126: control+U0126, RLIPC+U0126: remote liver ischemic preconditioning+U0126. B. p-ERK1/2-to-ERK1/2 ratio. n = 4 per group. ††P<0.01, †††P<0.001 versus RLIPC mice.

Furthermore, our western blot data showed that U0126 inhibited ERK1/2 phosphorylation in kidney tissue, in contrast to what was observed in individuals in the I/R group who did not receive U0126 treatment (*P<0*.*001*; Fig 9A and 9B). Taken together, these findings suggest that activation of ERK1/2 may mediate the renoprotective effect of RLIPC on renal I/R injury in mice.

## Discussion

In our present study, we for the first time discovered that RLIPC may significantly protect the kidneys against acute renal I/R injury. It effectively maintains renal function and preserves the structure of tubules while also reducing cell apoptosis, inflammation, and overall damage to the kidneys. Most importantly, our findings suggest that RLIPC may exert its protective effects via enhancing the phosphorylation of ERK1/2 after I/R.

Delayed graft function (DGF) and transplant kidney failure commonly occur after donor death and after standard donor transplantation due to I/R injury (IRI) during circulation before kidney transplantation surgery [22, 23]. Although there have been continuous research advancements, the molecular mechanism responsible for IRI has not been fully elucidated. In fact, there is a significant shortage and pressing need for targeted treatments that can address the fundamental pathophysiological features of IRI. This is also the reason why we conducted this study. By establishing an animal model of renal I/R, we examined the protective effects of RLIPC on mouse kidneys and, further, the underlying mechanisms of this intervention.

Studies in renal research have used several types of models of renal I/R, surgery-based animal models such as clamping of both renal pedicles (bilateral IRI), clamping of one renal pedicle (unilateral IRI), clamping of one or both renal arteries or veins, or unilateral IRI with contralateral nephrectomy (uIRIx) [24]. Here, we first established a mouse model of renal I/R according to our previous experience and previous literature, 45 min of ischemia followed by 24 h of reperfusion [12, 25], a classic model in this field. The creatinine and urea levels were successfully measured in our model and were consistent with the findings of other studies [25, 26]. Therefore, we tested the effect of RLIPC on kidneys. Fortunately, both creatinine and urea results were positive, which suggested the potential of this highly feasible intervention methods. Furthermore, kidney injury marker (NGAL) evaluation also provided evidence of the efficacy of RLIPC in mitigating I/R-induced renal dysfunction.

Renal IRI-induced AKI usually involves both functional and structural changes [1]. Therefore, we next conducted pathological staining of renal tissue to evaluate renal structural changes. Renal I/R can lead to both tubular and glomerular damage. Renal tubular damage includes swelling, necrosis, and shedding of tubular cells. This affects the normal function of the renal tubules, resulting in impaired urine production and excretion. On the other hand, glomerular damage, including glomerular mesangial cell proliferation and changes in the basement membrane, further worsens renal outcomes. These changes may lead to a loss of glomerular filtration function. In line with these findings, HE and PAS staining revealed dramatic renal tubular and glomerular damage after I/R, which was significantly alleviated after RLIPC treatment. Quantitative comparison of kidney damage scores revealed the same outcomes. These findings provide additional evidence for the renoprotective effect of RLIPC.

In addition, renal I/R can also trigger an inflammatory response, leading to inflammatory cell infiltration and the release of inflammatory factors in the renal interstitium. This may worsen kidney damage and affect the normal structure of the kidneys. Previous studies have investigated this topic and investigated various methods for alleviating renal I/R based on this mechanism [27–29]. Thus, we also tested inflammatory factors in our animal model to explore whether RLIPC also has the ability to alleviate the inflammatory response in kidney tissue. We selected IL-6 and TNF-α as inflammatory markers. The results showed that the expression of both of these genes increased dramatically in the CON group and decreased to a much lower level in the RLIPC group. These findings suggest that RLIPC may also protect kidneys by regulating the inflammatory response in the context of I/R injury. This finding also indicates the potential of RLIPC in other inflammatory mediator-related diseases in addition to renal I/R, although additional studies are needed to determine the underlying mechanisms involved before its final clinical translation.

With mitochondria emerging as pivotal players in this cellular death process due to their interactions with an extensive array of proteins associated with apoptosis [30, 31], apoptosis has been shown to be closely related to renal I/R injury [14, 32]. Accumulating evidence indicates that a set of proteins from the B-cell lymphoma (Bcl-2) family plays a significant role in governing neuronal death during cerebral ischemia. This protein family serves as a primary regulator of outer mitochondrial membrane permeability and has essential functions in the intrinsic apoptotic pathway. The release of cytochrome c into the cytosol, which initiates programmed cell death through apoptosis, is orchestrated by the Bcl-2 family. The mechanisms governing the release of cytochrome c and apoptosis mediated by cytochrome c are under the control of the Bcl-2 family [30]. Here, we demonstrated that RLIPC can significantly increase Bcl-2 while decreasing Bax expression in mouse kidneys subjected to I/R. These findings suggest that RLIPC may regulate mitochondrial function to alleviate cell apoptosis, although additional work is needed to verify this idea. We further performed a TUNEL assay and found similar results, with I/R-treated kidneys accompanying more apoptotic cells and RLIPC significantly reducing the percentage of apoptotic cells.

The reperfusion injury salvage kinase (RISK) and survivor activating factor enhancement (SAFE) signaling pathways are two widely examined cellular pathways involved in I/R injury [33–35], while the RISK pathway has been demonstrated to be the primary signaling pathway involved in IRI [36]. The members of the RISK pathway include protein kinase B (AKT), phosphoinositide 3-kinase (PI3K), extracellular-signal regulated kinase 1/2 (ERK1/2), rat sarcoma virus (RAS), serine/threonine kinase (RAF) and MAPK/ERK kinase 1/2 (MEK1/2) [33, 35]; in addition, ERK has been demonstrated to counteract the proapoptotic effects of Bcl-2 family members in the mitochondria by stabilizing the mPTP [37]. The SAFE pathway, on the other hand, mainly comprises Janus kinase (JAK) and signal transducer and activator of transcription-3 (STAT-3) [35]. We have shown that these pathways play essential roles in I/R injury, and strategies based on the regulation of related proteins are effective at protecting organs such as the heart [38, 39], kidney [12] and testis [40]. In this study, we measured the phosphorylation levels of AKT, ERK1/2 and STAT-3 to test the role of these two pathways in the RLIPC-mediated renoprotective effect. Our western blot analysis showed that there was a significant difference in the phosphorylation of ERK1/2 between the CON group and the RLIPC group, suggesting the intermediating role of ERK1/2, which was further supported by the results of immunohistochemical staining.

To further explain the underlying mechanisms behind the phosphorylation of ERK1/2, we applied U0126, a specific inhibitor of ERK1/2 phosphorylation, and performed experiments to assess renal function and injury levels. As predicted, treatment with the ERK1/2 inhibitor U0126 significantly counteracted the protective effect of RLIPC on mouse kidneys, consistent with the results for kidneys not treated with RLIPC. Inactivation of ERK1/2 by U0126 not only improved renal dysfunction but also reversed the morphological changes, inflammatory response, and renal cell apoptosis reduction effects induced by RLIPC. These results demonstrated the direct causal relationship between the activation of ERK1/2 and RLIPC-mediated renoprotective effects. This provided evidence for the potential of renal I/R treatment involving ERK1/2 activation as a target in the future, although randomized clinical trials are needed before final translation.

We acknowledge several limitations in our study. First, we tested only the renoprotective effects of RLIPC in an animal model. Human samples and cell research are lacking, which limits the translation of our conclusions to clinical settings and in-depth mechanistic exploration. Second, we investigated the role of only the RISK and SAFE pathways in RLIPC-mediated renal protection. Given the complicated nature of renal I/R occurrence and progression, other cellular processes, such as oxidative stress, ferroptosis and pyroptosis, are also worth investigating, as they were also shown to be closely related to renal I/R prevention and treatment. Also, we failed to evaluate antioxidant parameters, another more objective methods, in our experimental work, which is worthy of follow-up investigation. Finally, we investigated only the short-term effects of RLIPC on renal function and I/R-induced injury. However, whether it can also protect kidneys in the long term, such as by alleviating renal fibrosis, needs further evidence in the future, given that long-term IRI may lead to fibrosis and scarring of renal tissue, ultimately affecting the structure and function of the kidney.

In conclusion, the present study demonstrates that RLIPC protects the kidney against renal I/R injury, which may be associated with renal ERK1/2 phosphorylation. Furthermore, pharmacological inhibition of ERK1/2 phosphorylation with U0126 may abolish the renal benefits of RLIPC.

## Supporting information

S1 File The original uncropped and unadjusted images underlying all gel results reported in this submission. (PDF)
