# Supplementary material for: Remote liver ischemic preconditioning protects against renal ischemia/reperfusion injury via phosphorylation of extracellular signal-regulated kinases 1 and 2 in mice
Source: PLoS One. 2024 Aug 19;19(8):e0308977. doi: 10.1371/journal.pone.0308977 (PMC11332924; doi:10.1371/journal.pone.0308977)
Supplement: S1 File — (PDF) [file pone.0308977.s001.pdf]

In our study, we examined the expression levels of phosphorylated STAT-3, AKT and ERK1/2, and total STAT-3, AKT and ERK1/2 in renal tissue. All phosphorylated and total proteins have identical molecular weights and were probed on the same location of the same membrane. Initially, we assessed the expression of phosphorylated STAT-3, AKT and ERK1/2, after which we stripped the membrane and probed it for total proteins. The levels of phosphorylated proteins were then normalized using the levels of respective total proteins.

Figure 6A

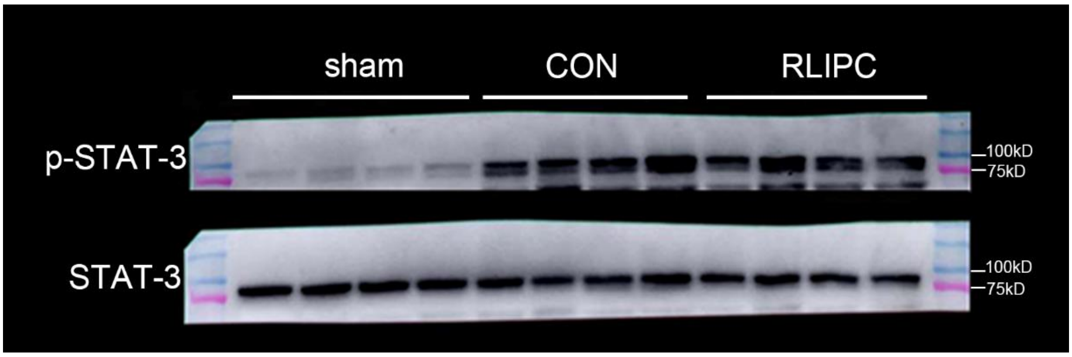

Figure 6B

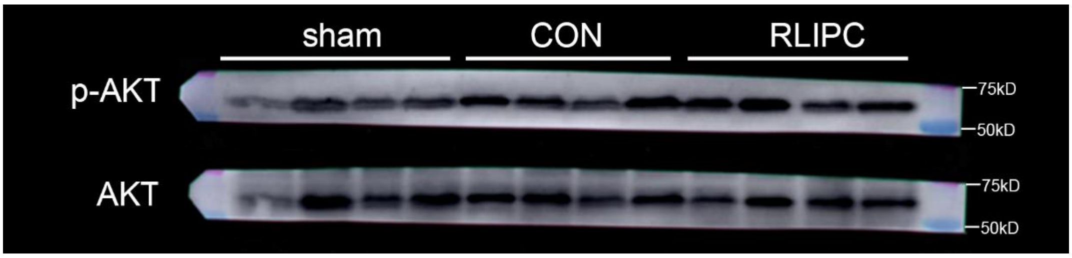

Figure 6C

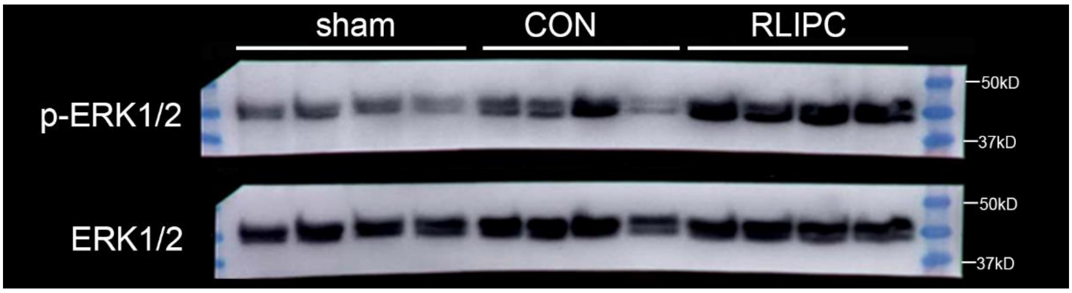
sham
CON
RLIPC
p-ERK1/2
ERK1/2
50kD
37kD

Figure 9A

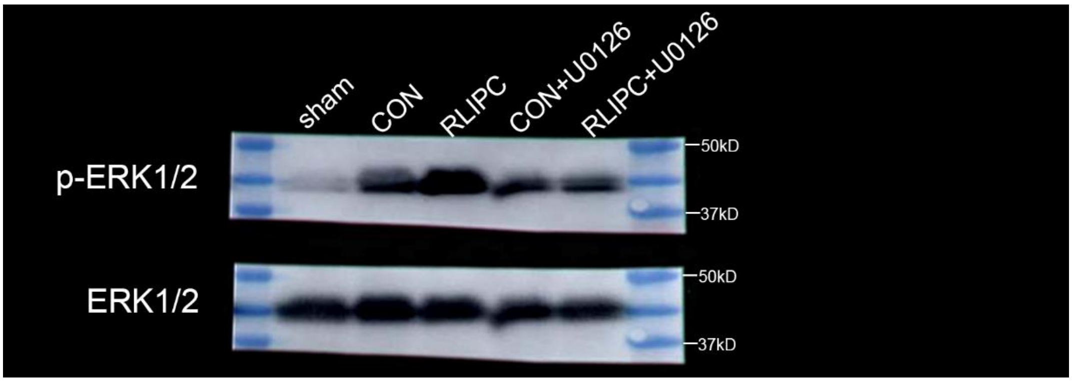
sham
CON
RLIPC
CON+U0126
RLIPC+U0126
p-ERK1/2
ERK1/2
50kD
37kD
